# The cellular microscopy phenotype ontology

**DOI:** 10.1186/s13326-016-0074-0

**Published:** 2016-05-18

**Authors:** Simon Jupp, James Malone, Tony Burdett, Jean-Karim Heriche, Eleanor Williams, Jan Ellenberg, Helen Parkinson, Gabriella Rustici

**Affiliations:** European Bioinformatics Institute (EMBL-EBI), European Molecular Biology Laboratory, Wellcome Trust Genome Campus, Hinxton Cambridge, CB10 1SD UK; European Molecular Biology Laboratory, Meyerhofstrasse 1, 69117 Heidelberg, Germany; Centre for Gene Regulation and Expression, University of Dundee, Dundee, DD1 5EH UK

**Keywords:** Ontology, OWL, Cellular phenotype, Imaging

## Abstract

**Background:**

Phenotypic data derived from high content screening is currently annotated using free-text, thus preventing the integration of independent datasets, including those generated in different biological domains, such as cell lines, mouse and human tissues.

**Description:**

We present the Cellular Microscopy Phenotype Ontology (CMPO), a species neutral ontology for describing phenotypic observations relating to the whole cell, cellular components, cellular processes and cell populations. CMPO is compatible with related ontology efforts, allowing for future cross-species integration of phenotypic data. CMPO was developed following a curator-driven approach where phenotype data were annotated by expert biologists following the Entity-Quality (EQ) pattern. These EQs were subsequently transformed into new CMPO terms following an established post composition process.

**Conclusion:**

CMPO is currently being utilized to annotate phenotypes associated with high content screening datasets stored in several image repositories including the Image Data Repository (IDR), MitoSys project database and the Cellular Phenotype Database to facilitate data browsing and discoverability.

## Introduction

Recent advances in imaging techniques make the study of complex biological systems feasible, particularly at the cellular level, complementing existing “omics” approaches, most notably genomics and proteomics, by resolving and quantifying spatio-temporal processes with single cell resolution [1]. High content screening (HCS) is an imaging based multi-parametric approach that allows the study of living cells. HCS is used in biological research and drug profiling, to identify substances, such as small molecules or RNA interference (RNAi) reagents, which can alter the phenotype of a cell. It can also be used to look at the effect of knocking out genes completely, or to determine protein localization by modifying genes to produce tagged proteins that can be visualized. Phenotypes may include morphological changes of a whole cell, or any of its cellular components, as well as alteration of cellular processes.

Correlative analysis of cellular phenotypes, specific to individual genes, with morphological imaging data from diseased tissue specimens (both human and mouse tissues) allow us to link phenotypic data to associated image annotations and metadata, leading to a powerful predictor of disease biomarkers as well as drug targets. For example, when a certain cellular phenotype, like ‘mitotic delay’ or ‘multi-nucleated cells’, observed in cells after gene knockdown experiments, is also observed in cells of a cancer tissue, this could give us an indication of which gene(s) might be involved in the aetiology of the disease, in that specific tissue. Knowledge of the functional implications of somatic tumor mutations can thus be used to design more targeted drug therapies.

Data derived from live cell imaging is typically associated with rich metadata, including genetic information, and can be more easily interpreted and linked to underlying molecular mechanisms. As we move to higher organisms, such as mouse and human, the degree of metadata available decreases (e.g. no genetic information is available for diseased human tissues), alongside the feasibility of assays that can be carried out in such organisms (e.g. genetic engineering is only possible in cell lines and mouse models). Taking this into consideration, it becomes evident that integrating imaging datasets from different biological domains could greatly advance our understanding of the molecular mechanisms underlying specific diseases.

Due to its late arrival on the “omics” scene, the imaging field has not yet achieved the same degree of standardization that other high-throughput approaches have already reached [1], thus hampering integration of image data with current biological knowledge. Standards are needed for describing, formatting, archiving and exchanging image data and associated metadata, including suitable nomenclatures and a minimal set of information for describing an imaging experiment. This is crucial to enable the establishment of databases and public repositories for image data and allow for the integration of independent datasets.

The use of ontologies to annotate data in the life sciences is now well established and provides a means for the semantic integration of independent datasets. Despite the availability of several species-specific ontologies for describing cellular phenotypes (e.g. the Fission Yeast Phenotype Ontology), there isn’t an appropriate infrastructure in place to support the large-scale annotation and integration of phenotypes across species and different biological domains.

As part of the BioMedBridges project,^1^ efforts are underway to integrate biological imaging datasets provided by emerging biomedical sciences research infrastructures, including Euro-BioImaging,^2^ for the provision of cellular image data; Infrafrontier,^3^ for mouse tissue image data, and BBMRI/EATRIS,^4^ for human tissue image data. Such infrastructures are generating a wealth of imaging data that can only be made interoperable through consistent annotation with appropriate ontologies.

There has been much work published on the development of cross-species phenotype ontologies and their benefits [2]. To date ontologies describing phenotypes exist for a host of species including mammalian phenotypes (MP; [3]), Ascomycetes (APO; [4]), *S. pombe* (FYPO; [5]) and *C. elegans* (WPO; [6]). There are also well established ontology design patterns for modeling phenotypes in a species and domain independent manner that utilise the Phenotype and Trait Ontology (PATO) [7]. These phenotypic descriptions are based around the Entity-Quality model (EQ) that refers to describing a phenotype in terms of an Entity (E), from one of many given reference ontologies, such as Gene Ontology (GO, [8]) and an associated Quality (Q), from PATO [9]. For example, a *“large nucleus”* phenotype could be expressed in EQ using the entity term *“nucleus”* [GO:0005634] from GO’s cellular component and the quality term *“increased size”* [PATO:0000586] from PATO. This model has been adopted by a range of model organism databases for the annotation of various phenotypes ranging from disease, anatomical and cellular phenotypes [10].

Ontology languages, such as the Web Ontology Language (OWL), allow us to express logical definitions for classes that describe class membership based on quantified relationships to other classes. The Basic Formal Ontology (BFO) defines the *“inheres in”* [BFO:0000023] relationship that can be used to capture the relationship between qualities, which in BFO are specifically dependent continuants, and the bearer of those qualities, which are typically independent continuants. For example, in order to logically define a *“large nucleus phenotype”* we say that the quality of *“increased size”* inheres in the bearer, which in this case would be the *“nucleus”.* We can express this relationship logically in OWL using existential quantification to assert that the class of all *“large nucleus phenotype”* is equivalent to the class of things that have an *“increased size”* quality that *“inheres in”* a *“nucleus”*. We could go on to further describe another class of phenotypes, such as a more general “nuclear size phenotype” and by virtue of the fact that *“increased size”* is a subclass of a more general *“size”* quality, use an OWL reasoner to automatically classify *“large nucleus phenotype”* as a subclass of *“nucleus size phenotype”*. Highly scalable reasoners, such as ELK [11], have made it practical for ontology engineers to fully exploit this expressivity when working with large ontologies. In the case of building phenotype ontologies, it means we can now build logical class definitions for a large number of phenotypes following the EQ pattern, and let the reasoner do the work to classify those phenotypes and infer equivalence across different phenotype ontologies.

A previous effort to develop a species neutral cellular phenotype ontology (CPO) was undertaken by Hoehndorf et al. [12]. The CPO was automatically generated and includes logical class definitions composed from GO and PATO terms. Whilst in principle this is a reasonable approach, in practice the resulting ontology was difficult to work with and did not provide a good vocabulary for data annotation. The size of the ontology coupled with limitations in standard ontology authoring software made it impractical to extend and maintain this ontology whilst keeping in sync with GO and PATO via the automatic generation process. The size and automatic label creation strategy also made it difficult for the biocurators to find terms for annotating data. It would have been a considerable amount of effort to manually clean the CPO to make it fit for purpose as a general annotation vocabulary for imaging datasets.

Our approach was therefore to build CMPO from the available data, using a post-composition approach where phenotypes were manually annotated with ontology terms that were later used to compose new stable phenotype terms in the ontology. These new terms were annotated with appropriate meta-data, such as synonyms and definitions that reflect how the terms are used in the data and literature.

## Results

**Fig. 1:**
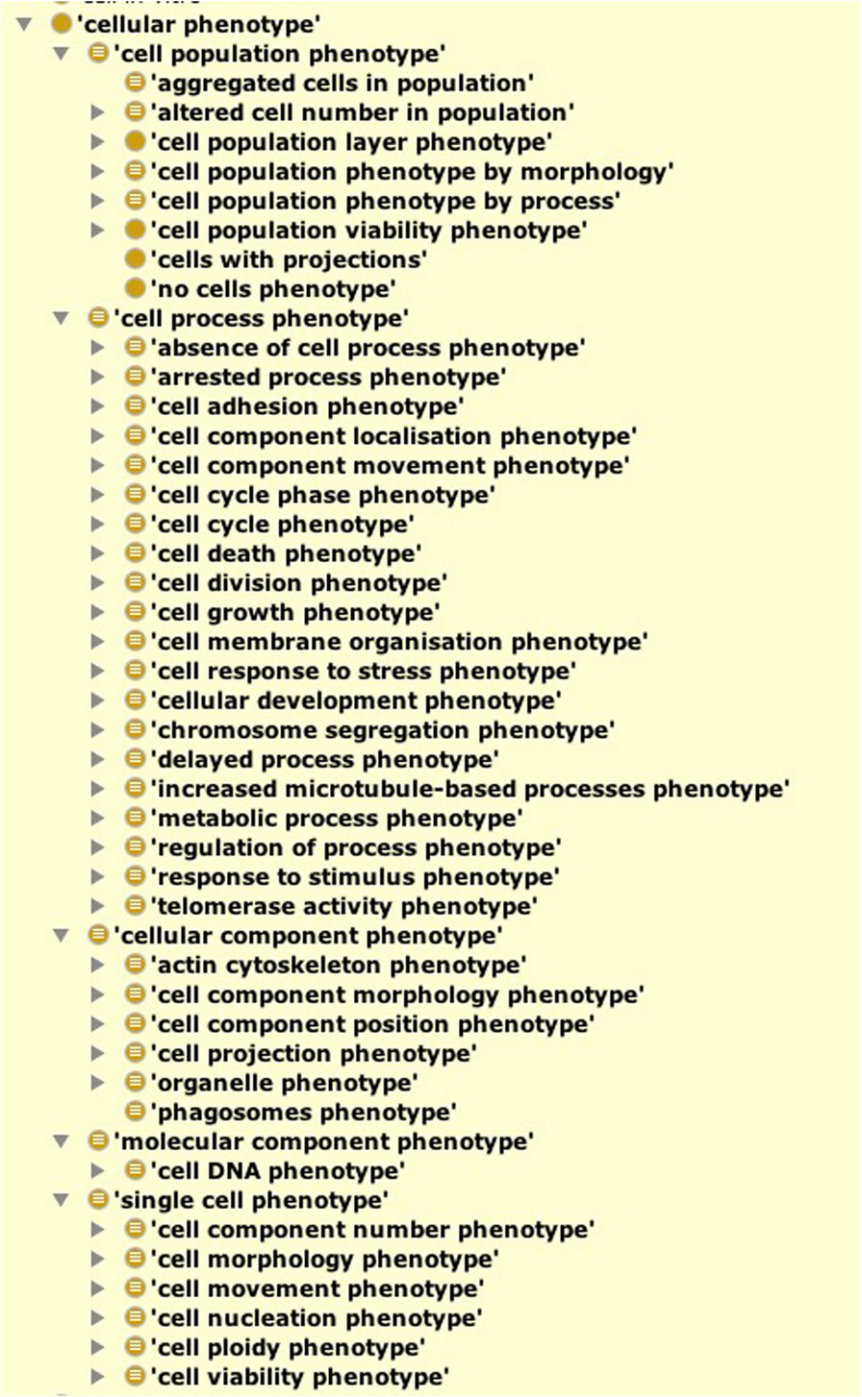
Visualisation of the top-level terms in the CMPO phenotype ontology showing cell process, single cell, cellular component and molecular component phenotypes

As of release 1.9 CMPO contains 361 phenotype terms. CMPO provides a root class called *‘cellular phenotype’* which is further divided into five major sub-types, namely; *‘cell process phenotype’, ‘cellular component phenotype’, ‘molecular component phenotype’*, *‘single cell phenotype’ and ‘cell population phenotype’.* (Fig. 1). Each of these categories represents a different level of granularity for which we see phenotype descriptions in the data. Every effort is made to ensure that each CMPO term has an equivalence axiom that describes the term using an OWL class expression. We strive to avoid asserting subclass axioms between named phenotype classes and instead use a reasoner to infer classification using logically defined classes.

## Cell process phenotype

The cell process phenotypes aim to capture phenotypic descriptions at the level of cellular processes. Using the Manchester OWL syntax (MOS) notation^5^ we can express a CMPO cell process phenotype as being logically equivalent to the anonymous OWL class *‘has_part some (‘process quality’ and (inheres_in some biological_process))’*, where the ‘*process quality’* comes from PATO and the *biological_process* term is from GO. In some cases, such as the CMPO ‘mitotic process phenotype’, we would like to capture all phenotypes that inhere either the GO *‘mitotic cell cycle’* or part of the GO *‘mitotic cell cycle’*. Whilst OWL provides the vocabulary for *union* (OR) operators in OWL class descriptions, this would take CMPO outside of the OWL-EL^6^ sublanguage. In order to keep CMPO within EL and have the ability to compute desirable subclass relations, we used two separate equivalence class axioms e.g. *‘mitotic process phenotype’* is equivalent to *‘has_part some (‘process quality’ and (inheres_in some (part_of some ‘mitotic cell cycle’)))’* and equivalent to *‘has_part some (‘process quality’ and (inheres_in some ‘mitotic cell cycle’))’*.

There are also cases where phenotype descriptions attempt to capture the absence of a process e.g. *‘absence of mitotic chromosome decondensation phenotype’*. Whilst PATO contains a quality called *‘lacking processual parts’*, it would be incorrect to assert that absence of mitotic chromosome decondensation is a quality that inheres in the mitotic chromosome decondensation process itself. To deal with such cases we make use of the BFO ontology *‘specifically depends on at all times’* [BFO:0000070] (also referred to as ‘*s depends on*’ or *‘towards’*) relation, that can be used to relate a relational quality or disposition to a relevant entity. For *‘absence of mitotic chromosome decondensation phenotype’* we describe it as a *‘lacking processual parts’* quality that inheres in the cell cycle as a whole, where the *‘lacking processual parts’* quality specifically depends on the ‘*mitotic chromosome decondensation phenotype’*. The fully qualified equivalent class description for this phenotype is ‘*has_part some (‘lacking processual parts’ and (towards some ‘mitotic chromosome decondensation’) and (inheres_in some ‘cell cycle’))’*. A similar pattern is used throughout CMPO to deal with cases of phenotypes where the phenotype describes the absence of a particular entity.

HCS data often includes phenotypes relating to protein localisation in the cell. CMPO aims to describe protein localisation phenotypes in terms of the protein localisation process that is occurring along with details of the protein complex being transported and in some cases the target and end location of the protein. Using the *‘transports or maintain localization of’* and *‘has target end location’* object properties from the OBO Relation Ontology we describe a complex phenotype as equivalent to *‘has_part some (‘occurrence quality’ and (inheres_in some (‘protein localization’ and (‘transports or maintains localization of’ some polypeptide) and (‘has target end location’ some cellular_component))))’.* The GO provides good coverage of protein localisation processes that CMPO has utilised to develop a branch of protein localisation phenotypes relating to various cellular components and the CMPO design pattern is consistent with the pattern used in the OWL edition of GO.

## Cellular component phenotypes

All cellular component phenotypes are logically described as any quality (non processual quality) that inheres in any cellular component from GO e.g. in MOS notation *‘has_part some (quality and (inheres_in some cellular_component))’*. Typically these observations relate to the morphology or position of a particular component in a cell. In order to drive all the necessary inference to infer subclasses of a general term such as *‘nuclear phenotype’* we describe these terms using three equivalence class axioms to capture qualities of the nucleus, nuclear parts, and any qualities that11 are *‘towards’* the nucleus.

## Molecular component phenotypes

The molecular component phenotype branch describes phenotypes at the level of molecules in the cell. All molecular component phenotypes are logically equivalent to *‘has_part some (quality and (inheres_in some ‘molecular entity’))’* where the molecular entity is a bio-molecule from the ChEBI ontology [13]. To date this branch of the CMPO only contains phenotypes terms relating to the shape of DNA molecules within the cell.

## Single cell phenotypes

Single cell phenotypes in CMPO describe phenotypes that are observed at the level of the whole cell. Single cell phenotypes are described as logically equivalent to ‘*has_part some (quality and (inheres_in some’cell in vitro’))’* where ‘cell in vitro’ is imported from the Cell Ontology [14]. The single cell phenotypes are further classified in terms of cellular component number, whole cell morphology, cell movement, cell nucleation and cell viability.

## Cell population

CMPO describes a cell population phenotype as a collection of qualities that inhere in a population of cells. We distinguish between qualities of the population as a whole and qualities of individual cells within the population using the Relation Ontology *‘bearer of’* relationship. For example, CMPO describes a *‘fewer mitotic metaphase cells’* phenotype as a *‘has fewer part of type’ quality that inheres in a population that* bears a *‘mitotic metaphase phenotype’*. In MOS we can define *‘fewer mitotic metaphase cells’* as equivalent to *‘cell population’ that ‘has_part some (‘has fewer parts of type’ and (‘bearer of’ some ‘mitotic metaphase phenotype’))’*.

## CMPO annotation properties

CMPO follows many standard conventions from the OBO foundry for ontology term metadata. Every CMPO term must have an rdfs:label and definition using the Information Artifact Ontology (IAO*) “definition”* [IAO:0000115] predicate. In cases of phenotype terms that could be traced back to a source publication or dataset, we used the *“definition source”* [IAO:0000119] predicate from IAO to link the term to the publication. The standard set of OBO synonym properties are also used to capture exact, broad and narrow synonyms for a term. The source CMPO OWL file imports the full GO and PATO ontology to support development of the ontology and to drive the inference. Finally we define a CMPO slim so that we can easily extract a simplified version of CMPO for a release of the ontology that exclude all the PATO and GO terms.

## CMPO availability

The CMPO homepage (http://www.ebi.ac.uk/cmpo) provides access to the ontology and issue tracker for submitting new term requests. The source ontology for CMPO is hosted on GitHub^7^ and it is also available from the NCBO BioPortal [15] and the EMBL-EBI’s Ontology Lookup Service (OLS).^8^

## Applications of CMPO

In the context of the BioMedBridges project, we want to demonstrate the power of interoperability of large-scale image data sets from different biological scales to enable drug target and biomarker discovery for human diseases, focusing on cancer as an example.

CMPO is being used to annotate mitotic phenotypes observed in live human cells, as well as cellular phenotypes from tissue microarrays of diseased tissues from both human patients and mouse models. Analysing phenotypic correlations between cellular and tissue data sets, and linking imaging data with molecular data, including the cancer genome sequence and expression data, will allow for in silico validation of the predictions and prioritization of biomarkers for validation in clinical research. In particular, we focus on genes with a function in controlling cell cycle and cell division, as well as invasive behaviour, for which comprehensive molecular and cellular datasets are available.

**Fig. 2:**
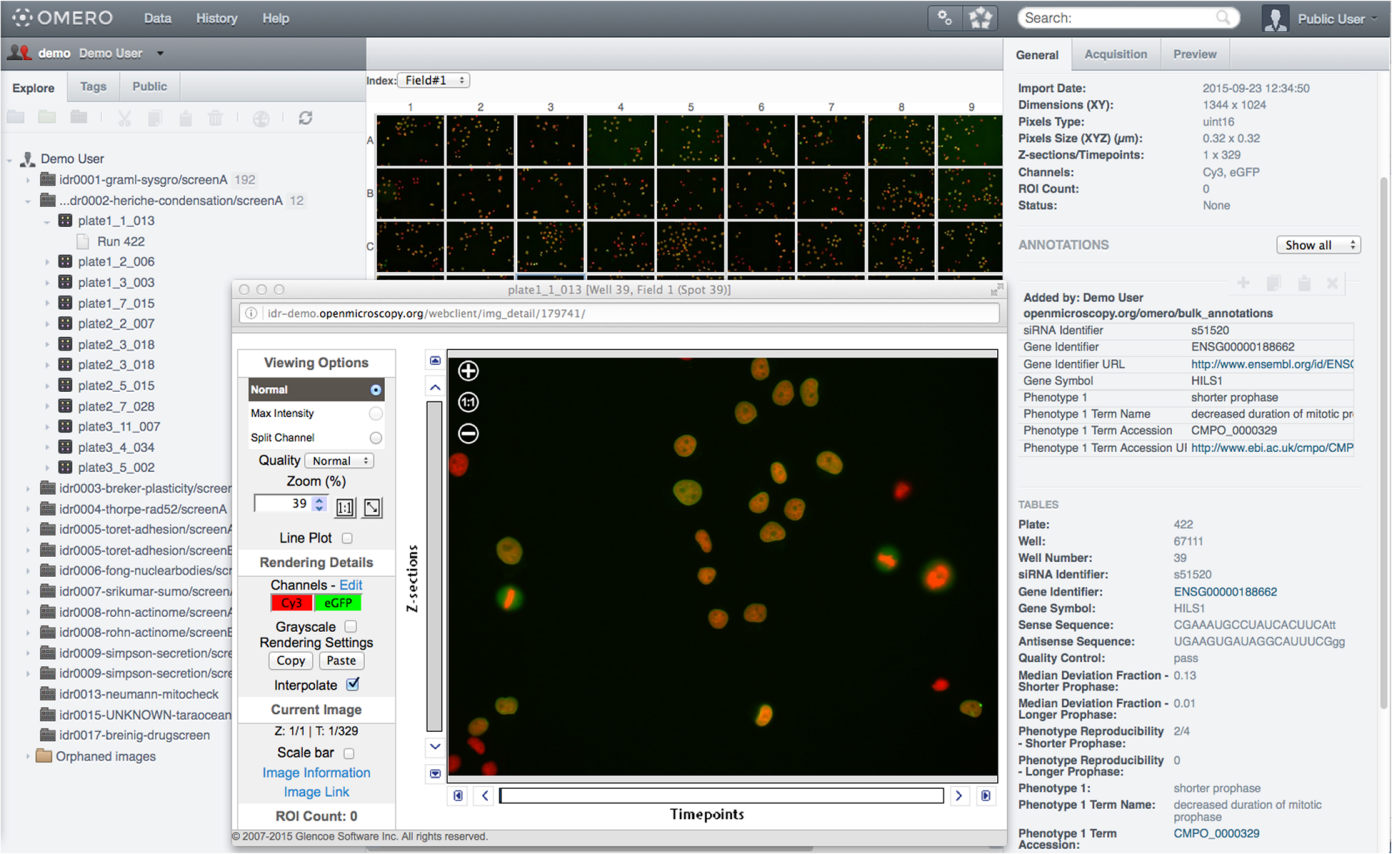
Screenshot of the Image Data Repository showing image meta-data that include phenotype annotation to CMPO term “decreased duration of mitotic prophase” [CMPO:0000329]

CMPO is currently being utilized to annotate phenotypes associated with HCS datasets stored in the Image Data Repository (IDR), a next generation repository currently being developed to: (i) provide easy access to ‘reference’ image data linked to peer-reviewed publications and support browsing, search and visualization of image data and metadata; (ii) facilitate the establishment and adoption of data standards to enable interoperability of image data; (iii) link such data to other biomolecular data resources (e.g. genomics databases, structural databases and functional annotation) and (iv) build a computational resource to support the re-analysis of image data and the development of new computational tools. IDR is built upon established, actively developed open source platforms and applications, including the OMERO software for visualization, management and analysis of biological microscope images [16]. The OMERO API is currently being extended to explicitly support ontological annotations and access CMPO through OLS to look up of additional information and subsumption queries [17]. Since CMPO has been applied to annotate phenotypes associated with IDR data (Fig. 2), 50 new phenotype terms have been added to the ontology. CMPO has also been integrated into the MitoSys project database^9^ and the Cellular Phenotype Database [18] to facilitate data browsing and discoverability. Work is in progress to add a functionality for ontology based browsing in CellCognition [19].

## Method

Eleven imaging datasets were initially sourced to collect a set of candidate phenotypic descriptions for manual ontology annotation [20–30]. Our approach was to annotate the phenotypes with terms from GO and PATO to generate EQ based annotations that would be later post-composed to form new CMPO terms. We developed a simple Web application called Phenotator for the data providers to submit and annotate their phenotypes with an EQ. The Phenotator is built using services from the NCBO BioPortal [15] to generate simple drop down menus and autocomplete search functionality to guide the users in generating EQs with appropriate terms (Fig. 1). Phenotator has a feature to export the collected EQ annotations as an OWL file containing new terms that are logically defined according to the SUBQ pattern,^10^ which can be expressed in Manchester OWL syntax as *“(has_part some (<Quality> and inheres_in some <Entity>))”.* One hundred twenty-seven phenotype descriptions from the original 11 datasets were entered into Phenotator, together with 41 phenotypes collected from cell migration assays (Z. Kam, personal communication) and 193 phenotypes from the GenomeRNAi database [31]. The domain experts entered EQ based descriptions for a total of 201 phenotypes.

The EQs were exported from Phenotator as an OWL file and loaded into the Protege OWL ontology editor. The generated OWL file imported the full Gene Ontology and PATO and the ELK reasoner was used to compute an automatic classification of the post-composed EQ terms. The biological curators and ontology experts were able to use this classification to both verify the collected EQs and inform the organisation of the upper level of the ontology so that the terms were classified into useful categories. After several iterations of this process, the post-composed terms were assigned permanent CMPO identifiers and relevant metadata for each term was collected in preparation for the initial release.

CMPO accepts new terms requests via the CMPO website and also accepts more structured term requests via the Webulous application. Webulous provides a service for specifying ontology term creation templates. These templates can be loaded into tools such as Google Sheets using the Webulous Google Sheets Add-on,^11^ so that users can submit batch requests of new terms to CMPO. The CMPO Webulous templates have been used by the Image Data Repository (IDR) curators as a mechanism for adding new terms to CMPO for both cellular process and cellular protein localisation phenotypes.

CMPO releases are managed using a continuous integration server and the OBO ontology release tool (Oort).^12^ CMPO is released as four files: a single OWL file that contains all axioms and the full GO and PATO import; a single Mireoted^13^ version of CMPO with only relevant GO and PATO terms, and two simple versions that only contain CMPO terms that are available in OWL or OBO format. All files are made public via the CMPO website and the CMPO GitHub repository.^14^

## Discussion

CMPO follows established best-practices from the Open Biomedical Ontology community and can provide a way to bridge low-level cellular phenotype data across species. Merging CMPO with other post-composed phenotype ontologies, such as FYPO, and classifying these together using a reasoner shows that equivalent terms can be inferred. Some manual intervention is required to harmonise the URIs used for some of the relationships and many terms don’t merge as expected because the OWL version of FYPO doesn’t use the SUBQ pattern used in CMPO. Best practices for the translations of EQ annotations into OWL statements are still emerging and inconsistent use of common OBO relationships and lack of shared design patterns suggest that there is still some work to be done to integrate the various cellular phenotype ontologies.

Most cellular phenotype ontologies contain terms for describing features such as cell size, shape and morphology that are often observations that can be considered subjective or are only valid in the context of a particular assay. For instance, nuclei are not bright unto themselves, but we have data where the phenotype has been recorded as “bright nuclei” in response to a particular treatment. CMPO currently includes terms such as “bright nuclear body” and “increased cell size”, however, these terms are unlikely to have a shared meaning across independent datasets. We believe having these terms in the ontology is important as they represent the vocabulary of the domain, but their use without additional context may be of less value for data integration. Ontologies for describing types of microscopy assays already exist and should be used in combination with ontologies like CMPO in order to provide a meaningful annotation, however, best practices and tooling to support this kind of structured data annotations are still lacking.

Despite the generality of the ontology building methodology applied, several challenges remain, including the lack of common design patterns that curators could consistently use when creating new terms, in the pre-composition phase. The need for common design patterns can be illustrated with an example from CMPO for the creation of an ‘increased cytoplasmic actin phenotype’ term. This term was initially problematic to annotate with a basic EQ because no term for cytosolic actin existed in GO. The curators initially used a close approximation which was EQ(‘actin filament’, ‘present in greater number in organism’), but the fact that the actin is localised to the cytosol is lost in the EQ. To increase the expressivity of the annotation in Phenotator a third column was added to capture additional modifiers to the EQ resulting in annotations emerging like EQE2 (‘actin filament’, ‘localised’, ‘cytosol’). There are other ways that one might consider describing this phenotype such as EQE2 (‘cytosol’, ‘has extra parts of type’, ‘actin filament’). Guidelines and tooling that help with guiding the curators to create a good EQ annotation are therefore needed to resolve ambiguities and develop a consistent strategy for creating new ontology terms.

Pattern-based tooling to rapidly generate new terms are emerging and these could nicely complement existing tooling that are primarily aimed at annotating phenotypes with EQs such as Phenotator and Phenote. Phenotator and Phenote do little to guide the annotator to make a correct EQ annotation and the translation of these annotations to OWL typically only allows for a basic SUBQ pattern. Tools like TermGenie [32] and Webulous offer greater flexibility for post composing terms, as they are not restricted to EQ alone and can use more expressive design patterns for the translation of input data into OWL.

## Conclusion

CMPO is a species neutral ontology for describing cellular phenotypes that has been established according to the best practices from the Open Biomedical Ontology community. This allows CMPO to be developed independently from other phenotype ontologies, but to also remain interoperable via inference derived from the use of logical class descriptions. This interoperability will allow future integration of data annotated with species-specific vocabularies with imaging data annotated with CMPO.

We are committed to the continued development of CMPO and the use of CMPO in tools such as CellCognition and resources such as the IDR, Mitosys and Mitocheck. We are developing better tools to support building ontologies from design patterns that allow us to engage the imaging user community in the future development of CMPO. Beyond the benefits in browsing and searching phenotypic data, CMPO also enables new data analysis. For example, by replacing free-text annotations, CMPO makes automatic evaluation of phenotypic similarity possible and allows systematic exploration of the links between gene function and loss of function phenotypes across experiments thus facilitating the conversion of phenotypic annotations to functional annotations. Additional work to harmonise the various cellular phenotypes ontologies with CMPO will provide new possibilities for integration and analysis of this kind of data across species.

## Footnotes

1 http://www.biomedbridges.eu/

2 http://www.eurobioimaging.eu/

3 https://www.infrafrontier.eu/

4 http://bbmri-eric.eu/

5 http://www.w3.org/TR/owl2-manchester-syntax/

6 http://www.w3.org/TR/owl2-profiles/#OWL_2_EL

7 https://github.com/EBISPOT/CMPO

8 http://www.ebi.ac.uk/ols

9 http://www.mitosys.org/

10 https://github.com/obophenotype/upheno/blob/master/docs/OWLAxiomatization.md

11 https://chrome.google.com/webstore

12 https://github.com/owlcollab/owltools

13 http://obi-ontology.org/page/MIREOT

14 https://github.com/EBISPOT/CMPO
